# Art in the abyss: creativity and the schizophrenic mind

**DOI:** 10.3389/fpsyt.2025.1617462

**Published:** 2025-06-26

**Authors:** Saranya T. S., Gayathri Raj, Kevimeno Kiso, Darisha Kharmih, Kiniholi Yepthomi, Sandeep Kumar Gupta, Gimcule Thou, Sidhanth Ashok, Ashalatha T. L., Arsha Maria Benno

**Affiliations:** Department of Psychology, CMR University, Bangalore, India

**Keywords:** artistic expression, art therapy, cognitive disorganization, creativity, psychosis, schizophrenia, therapeutic interventions

## 1 Introduction

Schizophrenia is, in general, a very complex and ultimately misunderstood psychotic disorder. A gross alteration in thought processes, perception, emotional regulation, and behavior characterizes it. This disorder affects 1% of the global population. This is the reason why most people are not aware of the symptoms, which consist of delusions, hallucinations, disorganized thinking, and cognitive deficits. These impairments will prevent most individuals from being able to function effectively in their daily lives. Therefore, such difficulties have been looked at from a clinical and pathological point of view for a long time.

Contrary to that, however, the fact that its debilitating nature has always been associated with some unusual forms of cognitive and creative expression (1) leads to a fundamental interrogation of the line separating genius and madness. Schizophrenia, creativity, and psychology, neuroscience, philosophy, and arts have enjoyed a great deal of discussion over time. From historical personalities like Vincent van Gogh and John Nash to contemporary artists and writers with psychotic disorders, the bond has captivated the interest of many.

Instead, it has been stated that divergent thinking, being the hallmark of creativity, is actually more pronounced in those predisposed to psychosis, affording them the ability to see correlations and ideas that evade common thinking (2, 3). This is not merely a theoretical condition. Psychotic schizophrenic art has uncovered the workings of common visual languages that defy conventional esthetic norms while opening new vistas to inner realities through art from schizophrenics, often called “Outsider Art” or “Art Brut.”‘

This paper seeks to examine the delicate nature of the schizophrenia-creativity axis. It considers how several altered cognitive states could aid an artistic production, ranging from hyper-associative thinking to a very reduced latent inhibition and strong emotions. Also addressed are the difficulties encountered in distinguishing disorganized thinking from meaningful creativity.

The paper further extends its discussion to a critical appraisal of schizophrenic visual art, its canon, features common among the various manifestations, and how it deviates from the traditional aesthetic frameworks. Through an interdisciplinary approach involving art history, psychology, and neuroscience, the study proposes an encompassing framework whereby a richer and more integrated comprehension of how schizophrenia conditions and is conditioned by artistic creation can be attained.

## 2 Theoretical background

Schizophrenia is manifested through a triad of symptom categories: positive symptoms such as hallucinations and delusions, negative symptoms such as apathy and withdrawal, and cognitive deficits (4). These impairments often restrict goal-directed behavior but may also dismantle rigid cognitive frames, potentially setting the stage for creative insight.

Hence, cognitive disorganization shuts down linear reasoning but fuels divergent thinking. This kind of thinking, therefore, allows schizophrenia not only to constrain but also to enable creative expression, depending on whether the person is able to make the necessary conceptual shift.

Creativity, being an agent of novelty and value, covers both divergent and convergent thinking processes. In schizophrenia, the divergent part is overdone and possibly to the detriment of forming coherent unions often referred to as convergence. Acting as irreconcilable opposites, it stands as one of the most basic stumbling blocks to any consensus-based interpretation of creativity.

## 3 Psychological mechanisms linking schizophrenia and creativity

Consider creativity from the abnormal development standpoint: one of the hallmark features of schizophrenia is cognitive disorganization, which might be considered to allow divergent thinking. Reduced inhibition of the prefrontal cortex is also seen in a creative thinker and a schizophrenic one; such decreased inhibition would allow freer associations but greatly increase incoherence (5, 6).

Hyper-associativity joins heterogeneous ideas in novel ways but veers into delusional thought if left unchecked (7). When functioning in an artistic manner, this process can render symbolic and metaphorical meaning, which is often expressed in such art forms as surrealism or abstraction.

Hallucinations and delusions, while agonizing, may have inspired symbolic metamorphosis. Auditory hallucinations have proven influential for composers; visual hallucinations shape schizophrenic visual art, often crystal clear with fragmentation, symbolism, and extreme abstraction (8).

Being emotionally hypersensitive may allow an artist to evoke emotional rawness in art that deals with solitude, existential angst, or transcendence. With the altered states of consciousness accompanying schizophrenia, the perception of reality is disrupted. On the contrary, it offers admission to internal mythologies or symbolic worlds. This friction between fragmentation and integration is chiefly articulated in the diagnosed artists’ art-marked by repetition, religious or cosmic rituals, and pictorial intricacies (9, 10).

## 4 What is schizophrenic art

Schizophrenic art situated in the wider field of Outsider Art or Art Brut refers to visual expression that comes from altered cognitive, emotional, and perceptual states of mind of persons with indigents of schizophrenia rather than external rules or art training. The art of this category cannot be defined solely by its pathology, nor can it be reduced simply as therapy. Rather, it is a distinct epistemological and symbolic system of representing psychotic experiences, such as hallucinations, delusions, and the disorganization of thought, into an understandable visual form (11, 12).

From a theoretical standpoint, schizophrenic art poses a challenge to the Cartesian split of rationality and creativity. The feature of this art-compulsive repetition; spiritual or cosmic imagery; bodies fragmented to another dimension; the juxtaposition of text and image-are not random manifestations of dysfunction but highly structured expressions of a peculiar perceptual logic (9). According to Jean Dubuffet, who coined the term Art Brut, these works are raw and naked expressions offering a direct access to deeper and perhaps even more primal layers of expression (12).

Reduced latent inhibition and associative thinking in schizophrenics in the neurocognitive domain may promote greater cognitive disorganization while simultaneously sustaining creative novelty (5). In visual art, this shows in an odd juxtaposition of seemingly unrelated elements, symbolic hyperdensity, and deviations from linear spatial logic. It resonates with hyper-associativity, by which very different ideas are linked in ways that are unconventional, yet symbolically meaningful (7).

Most importantly, schizophrenic art is rarely made for the sake of an audience or a commercial market; it is less about communicating an idea externally and more about achieving internal coherence-an organization of the chaos of psychotic experience into symbolic form (9). The extremely personal and usually solitary nature of its creation, therefore, makes it an honest articulation of altered subjectivity. Both the Prinzhorn Collection in Heidelberg and La Collection de l**’**Art Brut in Lausanne act as repositories of such expressions while helping to document the visual vocabularies of persons outside the mainstream academic and institutional traditions of art (11, 12).

It follows then that schizophrenic art should be seen not only as symptomatology of mental illness, but equally so as a genuine mode of thinking and expressing. It occupies an in-between space of pathology and poiesis, fully revealing the aesthetic capacity residing even in the most fragmentary modes of mental life.

## 5 Schizophrenic art: visual themes, canons, and divergence from normal aesthetics

Schizophrenic art, often demarcated within the boundaries of Art Brut or Outsider Art, possibly stands as a very special intersection between psychopathology and creativity. Schizophrenia is generally conceptualized as a state of disorder of perception, cognition, and affect. In contrast, its expressive forms, especially the visual forms, carry a distinct symbolic grammar and aesthetic scheme. The latter is shaped not simply by pathology; rather, it is the result of a peculiar reorganization of an inner experience into a visual language (11, 12). Fully, it stands outside the accepted academic or institutional frameworks; however, schizophrenic art is not to be regarded as simply arbitrary or chaotic as it follows its own code, while simultaneously marked by recognizable themes and formal formulae.

### 5.1 Visual themes and canonical features

Visual Themes are largely different and often deciphered psychopathology in schizophrenia art. While analysing the works of Vincent van Gogh who had a diagnosis of psychosis it can be found that his works deciphered distorted sense of reality and emotional turmoil and it was reflected using contrast colours (13).

Identity conflict, disintegration of self-image, psychological pain and conflict are also deciphered in many schizophrenic arts, self-portraits with bandaged ears by Vincent van Gogh (14), Nathan Waire ‘s self-portrait in Art- Brut collection reflects these visual themes.

There are certain recurrent canonical traits noted in schizophrenic art, despite the very individual character of the experiences produced by the illness. These are:

• Repetitive and compulsive detailing imitating cognitive perseveration and, at the same time, attempting to impose structure on internal fragmentation.
• Symbolic or spiritual imagery, coming from visions of religious, mythic, or cosmic nature, suggestive of delusional content or transcendental themes.
• Fragmented bodily forms and disoriented spatial organization conveying the disruption of body schema and thought disorder characteristic of psychosis.
• Text-image fusion-methods in which words become embedded into or intertwined with images, standing for linguistic disorganization and symbolic condensation.
• Isolation from prevailing artistic trends, deep personal visual systems contrived with utter disregard for any extrinsic validation of aesthetic judgments (7–9).

These motifs, therefore, point toward an ironic sort of canon of the non-canonical into which, from similar neurocognitive and affective disturbances, a communal visual language is shaped. Collections such as the ones curated by Hans Prinzhorn in early 20th century Germany and Jean Dubuffet’s Collection de l’Art Brut in Lausanne attest to the recurrence of these features across geographies and generations (11, 12).

### 5.2 Divergence from normal aesthetics

The fundamental essence that distinguishes schizophrenic art from mainstream aesthetic traditions is not only its thematic focus but also its epistemological orientation. Traditional or “normal” aesthetics operate on a communicative paradigm: the artist is producing for the audience, all within culturally legible frameworks that involve conventions of composition, symbolism, and perspective to divide meaning or give shared understanding. Conversely, schizophrenic art develops mainly as private cosmology, a symbolic system directed inward, sideways, not for interpretation but for psychic survival or internal coherence (Dubuffet, as cited in Cardinal, 12; Wango, 10).

In addition, the schizophrenic visual artworks rarely orient themselves toward formal composition, opting instead to impose a hyper-associative order in place of typical linear narrative or spatial considerations that align with the fragmented nature of schizophrenic thinking: visual compositions appear densely packed, disorganized, and fairly affective-intensity bearing; whereas such characteristics probably run contrary to regular aesthetic canons but by-and-large comprehend psychological logic (5, 7).

This difference is not viewed as a limitation but as two distinct modalities of cognition. Diminuted latent inhibition brought in by schizophrenic tendency enables unusual association of ideas and forms that in a lesser pathological context could also spur creativity (3). In schizophrenic art, on the other hand, such associations tend to create visual imagery of an overloaded or incoherent nature: “madness” in the eyes of conventional viewers, which are clothed symptoms and narrated aesthetic forms.

Hence, the separation from normative aesthetics inflicts a divorce in terms of form and content but also accrue in intention and ontology. Schizophrenic art is often not created to be shared, bought, or judged aesthetically-it-forced out of the compulsion to externalize inner chaos, to inscribe meaning on perceptual fragmentation, and to render psychotic experience visible.

## 6 The use of art in therapy

In schizophrenia, art therapy expresses the inner environment when language fails. Visual arts act as an alternative language for the projection of hallucinations, delusions, and fragmented feelings into a relatively coherent form. Music and writing therapy promote emotional integration and narrative coherence in a similar way. This happens even more-so in nonverbal or acute psychotic behavior, during which standard talk therapy has very little offer (15).

In clinical studies (16), the patients’ paintings gradually show more organization and symbolism with steady therapeutic work, which points toward the processing of emotion and integration of cognition. The earliest developments of Edward Adamson at Netherne Hospital in the UK prefaced the concept of healing through non-directive art-making.

### 6.1 Theoretical foundations: from pathology to potential

Art therapy reconceptualizes schizophrenia as not simply a site of pathology but rather a reservoir of symbolic imagination. Based on Donald Winnicott’s (17) idea of a “potential space”—as an in-between realm of reality and fantasy-art becomes a realm wherein schizophrenic disintegration can find reassembly through acts of play and creativity.

In a similar vein, the Jungian notion of active imagination can inform this. The act of creating art allows one to work directly with unconscious content and bypass damaged functions of the ego. It is, then, a therapeutic approach that does not seek to stop madness but rather to symbolize it and to give patients the power over their internal mythologies (18).

### 6.2 Art as a therapeutic medium in schizophrenia

Art therapy forms a rare radioactive agent for treatment of expressive and emotional disturbances in schizophrenia. It utilizes the method of nonverbal art expression-mostly visual art to facilitate the integration of cognition and affect in people who have experienced incoherence in their speech as a result of psychosis. Because this flattens affect and obscures formalized thought processes in schizophrenia, most of the standard psychotherapeutic approaches fail to gain access to the patient**’**s subjective experience. Art, however, works past these conditions and establishes a symbolic language that expresses internal states (16).

An individual with schizophrenia experiences the emergence of hallucinatory, delusional, and affect-disorganizing inner stimuli. Production, in the psychotherapeutic sense, transforms these chaotic inner stimuli into structured aesthetic forms. Creation then reduces the psyche**’**s agony while reconstructing identity and emotional firmness. Dutchman Edward Adamson was a significant activist for promoting his theory of patient art through the non-directive method in Netherne Hospital, Surrey, UK, by giving patients total freedom to choose how they wanted to work with art materials. His studies concluded that therapeutic art was not merely a channel for evidence for diagnosis but could become a way of psychological healing and expression of the self (9).

### 6.3 Reconceptualizing psychopathology through art

Art therapy for schizophrenia confronts the pathologizing gaze of psychiatry by assigning to the artistic act a far more noble function than mere symptom expression: namely, that of self-reconstruction. The therapeutic element lies in transforming an individual’s fragmented perceptual and emotional world into legible symbolic forms. In this respect, art becomes a transitional space in the spirit of Winnicott’s (17) idea of “potential space” in which inner and outer realities may be creatively mediated.

It is also a conduit for what Jung calls “active imagination”-a psycho-dynamic process in which the unconscious can become accessible through creative production. For schizophrenic ego boundaries often being compromised, art provides a bounded, aesthetic field in which fragmentation inherent in self-representations can be reintegrated. (18)

## 7 Neuropsychological and cognitive dimensions

Neurocognitively, schizophrenia exhibits hypofrontality, deficits in working memory, and dysregulated dopaminergic activity that compromise executive function but might also contribute to hyper-associativity, a major mechanism in creativity (5). In excess, reduction in latent inhibition and disinhibited semantic networks can be detrimental; on the other hand, at milder levels, they can aid in the formation of novel idea connections-the very basis of symbolic and visual creativity.

In visual-art therapy, psychologically relevant features might be manifested in: Fragmented spatial logic (representation of thought disorder).

• Symbolic religious or cosmic imagery
• Repetitive detailing (reflecting compulsive cognitive patterns)
• Text-image fusion (in response to linguistic disorganization)

This category of psycho-visual phenomena is not a random error of transmission between internal and external realities but instead represents the logic of psychosis in visual-symbolic terms.

### 7.1 Clinical application and modality-specific benefits

• Visual Art Therapies: It allows symbolic projection of patient delusions and hallucination into metaphor. The themes often attempted in Art Therapy are cosmologies, sacred geometries, or distorted anatomies—each providing a glimpse into the psychotic landscape of the patient.

• Narrative and Writing Therapy: Although less researched in schizophrenia, writing therapy is considered to promote temporal integration with the patient in reclaiming disrupted autobiographical memory and narrative identity (19).

• Music Therapy: They bypass emotional and engagement-related memory circuits. Especially with negative symptom clusters (e.g., anhedonia, alogia), music provides affective resonance prior to verbal access (20).

• Group Art Therapy: Social mirroring and reduction of isolation are important factors in allowing for interpersonal feedback within a safe symbolic frame, a function crucial given the characteristic social withdrawal with schizophrenia.

• Emotional Regulation and Affective Catharsis: Art creation activates limbic sites of affect and memory, enabling pent-up feelings inaccessible solely through speech (20).

• Cognitive Integration: Longitudinal studies show that artworks made in therapeutic settings tend to progress from disorganized imagery to organized and symbolically potently-loaded visuals—signifying neural reorganization along with cognitive integration (15).

### 7.2 Clinical evidence and case examples

Clinical studies confirm the therapeutic potential of art in schizophrenia. The study of Hanevik et al. (16) argued that through continuous engagement in art therapy, the artworks developed increasing levels of coherence and emotional symbolism similar to the psychological improvement. It was the work of Adamson that served as a watershed moment in British psychiatric hospitals and development of art therapy. He found that nondirective art-making, in which the patient chooses the medium, content, and style, engenders psychological autonomy and dignity (21).

Uttley et al. in a systematic review argue that art therapy improves global functioning and emotional wellbeing, especially on occasions when verbal therapies fall short. Music therapy is considered to be helpful with treating negative symptoms for instance anhedonia or avolition (20).

## 8 Conclusion

The side by side relationship of schizophrenia and creativity by itself, refuses that clinical pathologization or romantic idealization take hold. Rather than being an actual manifestation of disorganized thinking, the artistic expressions of schizophrenics reveal rich, symbol-laden inner worlds that stand outside conventional norms and provide profound insights into the workings of the human mind. Schizophrenic art is considered justifiably categorized in accord with that portion of art labeled “Outsider Art” or “Art Brut,” always held as a testimony to the creative potential in differing states of mind. Its opposite features-repetitive compulsion, broken symbolism, astral imagery, and fusion of text and image-do not represent aesthetic failure but rather an ictal alternate logic.

By examining some of these psychological working mechanisms-hyper-associativity, reduced latent inhibition, and emotional hypersensitivity-we get a picture of how cognizance disorganization may dismantle linear reasoning while uniquely fostering creative insight. The works of schizophrenic artists are not just diagnostically referable artifacts but meaningful creations articulating inner mythologies, spiritual tensions, and perceptual fragmentation.

To give these expressions their artistic legitimacy enables a more sympathetic and even deeper understanding of schizophrenia-not as a disorder but as another way of seeing and rendering reality. It respects that creativity, however unconventional, becomes, for those dissipated in the abyss of psychosis, a life preserver and a bridge across that abyss.
